# Stage-Specific Transcriptomic Analysis Reveals Molecular Basis of Ovarian Sterility in Triploid Turbot (*Scophthalmus maximus*)

**DOI:** 10.3390/ani16091357

**Published:** 2026-04-28

**Authors:** Xiaoxuan Sun, Lifang Li, Luyao Cheng, Zhen Meng, Wenteng Xu, Xinfu Liu

**Affiliations:** 1 State Key Laboratory of Mariculture Biobreeding and Sustainable Goods, Yellow Sea Fisheries Research Institute, Chinese Academy of Fishery Sciences, Qingdao 266071, China; xiyi7941@foxmail.com (X.S.); llf846225@163.com (L.L.); chengly61@163.com (L.C.); xuwt@ysfri.ac.cn (W.X.); liuxf@ysfri.ac.cn (X.L.); 2 College of Fisheries and Life Sciences, Shanghai Ocean University, Shanghai 201306, China; 3 College of National Engineering Research Center for Marine Aquaculture, Zhejiang Ocean University, Zhoushan 316022, China

**Keywords:** turbot, *Scophthalmus maximus*, triploid, gonad development, sterility, transcriptome analysis

## Abstract

Triploid turbot (*Scophthalmus maximus*) exhibit superior growth and survival, yet the molecular basis of their sterility—a key trait for aquaculture—remains largely unexplored. This study investigated ovarian development and transcriptomic profiles in diploid and triploid *S. maximus* at three key stages (6, 10, and 20 months post-hatch, mph) to elucidate the stage-specific molecular mechanisms underlying triploid sterility. Histological analysis revealed that diploid ovaries progressed through normal oogenesis to the early vitellogenic stage by 20 mph, whereas triploid ovaries were arrested at the oogonial stage, with only occasional primary oocytes and extensive connective tissue infiltration. Comparative transcriptomic analysis identified 13,305, 14,599, and 13,331 differentially expressed genes (DEGs) between triploid and diploid ovaries at 6, 10, and 20 mph, respectively. Functional enrichment analysis showed that DEGs were significantly associated with meiotic processes, cell cycle regulation, energy metabolism, and apoptosis. Key meiotic genes (*spo11*, *dmc1*, *sycp3*) were consistently upregulated in triploids across all stages, while the DNA repair gene *rad51* was paradoxically downregulated, indicating attempted but aberrant meiotic initiation. Oogenesis regulators (*gdf9*, *bmp15*, *pou5f3*) and energy metabolism genes (*ndufa11*, *sdha*, *cox5a*) were significantly downregulated, whereas apoptosis-related genes (*eif2ak3*, *apaf1*) were upregulated. Notably, KEGG pathway analysis revealed stage-specific shifts from stress-induced apoptosis and p53 signaling at early stages to proteasome activation at later stages, suggesting a transition from active germ cell elimination to maintenance of cellular homeostasis in developmentally arrested ovaries. Collectively, these findings demonstrate that triploid sterility is associated with coordinated dysregulation of meiotic progression, metabolic, and apoptotic pathways, providing a high-resolution molecular framework for understanding reproductive failure in triploid fish and informing strategies for optimizing triploid production in aquaculture.

## Simple Summary

Turbot (*Scophthalmus maximus*) is a commercially important farmed fish, but normal reproductive development consumes substantial energy, reducing growth and survival. By producing sterile triploid fish, which possess three sets of chromosomes, this energy is redirected toward growth, conferring significant benefits for aquaculture. However, the molecular mechanisms underlying triploid sterility remain poorly understood. In this study, we examined ovarian development in diploid (normal) and triploid (sterile) turbot at three key stages (6, 10, and 20 months after hatching). Histological analysis revealed that triploid ovaries failed to develop normally, with eggs unable to mature. By analyzing gene activity, we identified widespread disruptions in processes essential for egg formation, energy production, and cell survival, ultimately causing ovarian underdevelopment. Notably, we observed a shift from early elimination of defective germ cells to later maintenance of cellular stability. These findings explain why triploid turbot are sterile and provide a valuable foundation for improving triploid production efficiency in aquaculture, supporting more sustainable fish farming practices.

## 1. Introduction

Polyploidy breeding is a cornerstone strategy for the genetic improvement of aquaculture species. By manipulating ploidy levels, this approach can enhance economic traits and environmental adaptability, with triploid induction being the most extensively applied method to date [1,2,3]. In triploids, the presence of an extra set of chromosomes disrupts normal meiotic segregation, leading to gonadal dysgenesis and sterility. This sterility confers two key advantages. First, it allows fish to redirect energy from reproduction to somatic growth, thereby mitigating the quality deterioration, growth stasis, and increased mortality associated with sexual maturation in diploids. Second, it provides reproductive containment, preventing genetic pollution of wild stocks from escaped farmed fish and safeguarding natural germplasm resources [4,5,6,7]. Owing to these benefits, triploid induction has been successfully commercialized in various species, including common carp *Cyprinus carpio* [8], common bream *Abramis brama* [9], Atlantic salmon *Salmo salar* [10], and olive flounder *Paralichthys olivaceus* [11].

A consistent phenotype across triploid teleosts is abnormal gonadal development. Histological studies have revealed that oogenesis in female triploids is universally arrested, with oocytes failing to reach maturity. For instance, female triploid red crucian carp *Carassius auratus* red var. produce only cystic oogonia and a few primary oocytes [12,13]. Similarly, primary oocytes are absent in triploid female rainbow trout *Oncorhynchus mykiss* [14], while in the Indian catfish *Heteropneustes fossilis*, oocyte development is arrested with reduced ovarian volume [15]. In extreme cases, such as in artificially induced triploid *P. olivaceus*, ovarian development is severely impaired, exhibiting atrophy and testicularization [16]. These observations confirm that sterility in female triploids is a conserved phenomenon rooted in meiotic failure.

Complementing these histological findings, molecular studies have begun to unravel the gene expression changes underpinning triploid sterility. In female triploid Pacific oysters *Crassostrea gigas*, sterility has been linked to the downregulation of mitotic cell cycle genes [17]. In triploid *C. auratus* red var., reduced *gthr* expression is thought to impair gonadotropin signaling and sex hormone synthesis. In triploid hybrid groupers (*Epinephelus coioides*♀ × *E. lanceolatus*♂), delayed ovarian development is associated with upregulation of steroidogenesis genes and downregulation of key oogenic regulators such as *gdf9*, *bmp15*, and *pou5f1* [18]. Furthermore, in triploid *P. olivaceus*, upregulation of testis-related genes including *amh*, *amhr2*, and *wnt4* correlates with the ovarian testicularization phenotype [16]. While these studies offer valuable insights, they often provide a snapshot of gene expression at a single time point, underscoring the need for comprehensive, stage-specific investigations into the molecular dynamics of triploid gonadal development.

Turbot (*Scophthalmus maximus*) is a commercially valuable marine fish in which triploidy confers significant production advantages. Triploid *S. maximus* exhibits significantly higher growth rates and an 8% increase in survival rate compared to diploids at a given age, particularly following sexual maturity [19]. These benefits arise because sterility prevents the energy diversion and physiological stress associated with gonadal development, thereby mitigating maturation-induced growth retardation and mortality. To harness these advantages, extensive efforts have been devoted to developing triploid induction protocols, resulting in a method now applicable at a commercial scale [20,21,22]. Critically, female triploids are sterile due to complete meiotic arrest, with histological analyses revealing that oogenesis is arrested at an early stage, with few oocytes progressing beyond the oogonial stage [23]. However, the molecular regulatory mechanisms driving this gonadal hypoplasia remain largely elusive, limiting our ability to fully understand and potentially manage triploid sterility in this species.

To address this knowledge gap, the present study adopts a developmental approach. These stages were selected to represent key transitions: meiotic stage (6 mph), previtellogenic growth (10 mph), and early vitellogenesis (20 mph), thereby enabling stage-specific dissection of molecular events leading to triploid sterility [24,25,26]. We collected ovarian tissues from diploid and artificially induced triploid *S. maximus* at these three stages (6, 10, and 20 mph) for histological observation and comparative transcriptomic analysis. By characterizing stage-specific gene expression profiles, this study aims to elucidate the molecular mechanisms underlying the sterility of female triploid *S. maximus*, thereby providing novel insights into the differential regulatory networks governing fertility in diploids and sterility in triploids.

## 2. Materials and Methods

### 2.1. Generation and Verification of Experimental Fish

Triploid *S. maximus* were artificially induced from fertilized eggs using a hydrostatic pressure shock protocol as previously described [22]. Briefly, fertilization and incubation were conducted at 14.5 ± 0.5 °C. At 5.5 min post-fertilization, eggs were subjected to hydrostatic pressure treatment at 60 MPa for 6 min to induce triploidy. Diploid controls were full-siblings derived from the same parental cross, generated from the same batch of fertilized eggs without pressure treatment. Diploid and triploid fish were reared separately under identical conditions at the Ganyu Research Center of East China Sea Fisheries Research Institute (Ganyu, China). Aquaculture conditions were strictly controlled as follows: salinity 28.6, pH 7.8, dissolved oxygen > 7.8 mg/L, water temperature 18 ± 1 °C, light intensity 300 lx, and a photoperiod of 16 h light: 8 h dark.

Prior to tissue collection, ploidy was verified for each sampled fish. A small piece of gill filament was collected, gently homogenized, stained with DAPI (0.6 μg/mL), and filtered through a 300-mesh nylon screen. Relative DNA content was analyzed using a CytoFLEX flow cytometer (Beckman Coulter, Brea, CA, USA), with diploid samples from corresponding stages serving as internal controls. Individuals exhibiting approximately 1.5 times the DNA content of diploid controls were confirmed as triploid. Only fish with confirmed ploidy status were used in subsequent analyses.

### 2.2. Fish Sampling and Tissue Collection

At each developmental stage (6, 10, and 20 months post-hatch, mph), female fish were randomly selected and anesthetized with MS-222 (20 mg/L; Solarbio, Beijing, China).

Following ploidy confirmation, ovarian tissues were rapidly dissected from each confirmed triploid female and from diploid controls. Tissues were divided into two portions: one portion was flash-frozen in liquid nitrogen and stored at −80 °C for RNA extraction, and the other was fixed in Bouin’s solution for histological examination.

### 2.3. Histological Analysis

Ovarian tissues fixed in Bouin’s fixative for 24 h were transferred to 70% ethanol and stored until processing. Histological sections were prepared following the method described in previous studies on *S. maximus* gonadal histology by Meng et al. [27]. Samples were dehydrated through a graded ethanol series, cleared in xylene, and embedded in paraffin wax. Serial sections of 5 μm thickness were cut using a microtome, deparaffinized, and stained with hematoxylin and eosin (HE). Stained sections were observed under a Nikon EClIPSE 80i microscope (Nikon Japan, Tokyo, Japan) and photographed. Oocyte development was classified into stages according to previously established criteria for *S. maximus* [24]: phase I (primary oocytes/perinucleolar stage), phase II (previtellogenic oocytes/cortical alveoli stage), and phase III (early vitellogenic oocytes with yolk granule accumulation).

### 2.4. RNA Extraction, Library Construction, and Sequencing

Ovarian tissues were collected from three confirmed female individuals per ploidy group (diploid and triploid) at each developmental stage (6, 10, and 20 mph), resulting in 18 samples. Sample designations were: D6-1-3 (diploid, 6 mph), T6-1-3 (triploid, 6 mph), D10-1-3 (diploid, 10 mph), T10-1-3 (triploid, 10 mph), D20-1-3 (diploid, 20 mph), and T20-1-3 (triploid, 20 mph).

Total RNA was extracted from each ovarian tissue sample using the RNAprep Pure Animal Tissue Total RNA Extraction Kit (Tiangen, DP431, Beijing, China) following the manufacturer’s instructions. On-column DNase I digestion was performed at room temperature for 15 min according to the manufacturer’s protocol. RNA concentration and purity were measured using a NanoDrop ND-2000 spectrophotometer (GE, Wilmington, DE, USA). RNA integrity number (RIN) was determined using an Agilent 2100 Bioanalyzer Lab-on-chip System (Agilent Technologies, Santa Clara, CA, USA). Only samples with RIN ≥ 8, A260/A280 ratio between 1.8 and 2.2, and A260/A230 ratio ≥ 2.0 were used for subsequent library construction.

Sequencing libraries were constructed from 1 μg of total RNA per sample using the Illumina Stranded mRNA Prep Ligation Kit (Illumina, San Diego, CA, USA) according to the manufacturer’s protocol. Poly(A) + RNA selection, fragmentation, double-stranded cDNA synthesis (SuperScript Double-Stranded cDNA Synthesis Kit, Invitrogen, 11917010, Thermo Fisher Scientific, Waltham, MA, USA), adapter ligation, and PCR amplification were performed as specified. Library quality and concentration were assessed on an Agilent 2100 Bioanalyzer. Paired-end sequencing (2 × 150 bp) was conducted on an Illumina NovaSeq X Plus platform by Majorbio Biotechnology Co., Ltd. (Shanghai, China).

### 2.5. Transcriptome Assembly and Functional Annotation

Raw sequencing reads were processed using fastp software (v0.23.4, https://github.com/OpenGene/fastp (accessed on 10 October 2025)) to remove adapter sequences, low-quality bases (Phred quality score ≤ 10 for >20% of bases), and reads with ambiguous base (N) content exceeding 5%. Clean reads were aligned to the *S. maximus* reference genome using HISAT2 (v2.1.0). Transcript assembly and read count quantification were performed using StringTie. Gene expression levels were normalized as transcripts per million (TPM).

Functional annotation of assembled unigenes was conducted by Majorbio Bio-pharm Technology Co., Ltd. (Shanghai, China) using DIAMOND software (v2.1.9, https://github.com/bbuchfink/diamond (accessed on 10 October 2025)) against multiple public databases: SWISS-PROT (http://web.expasy.org/docs/swiss-prot_guideline.html (accessed on 10 October 2025)), NR (https://www.ncbi.nlm.nih.gov (accessed on 10 October 2025)), GO (http://www.geneontology.org (accessed on 10 October 2025)), KEGG (http://www.genome.jp/kegg/ (accessed on 10 October 2025)), eggNOG (http://eggnogdb.embl.de/ (accessed on 10 October 2025)), and Pfam (http://pfam.xfam.org/ (accessed on 10 October 2025)).

### 2.6. Differential Gene Expression Analysis

Differentially expressed genes (DEGs) between diploid and triploid groups at each developmental stage were identified using DESeq2 software (v1.42.0, https://bioconductor.org/packages/release/bioc/html/DESeq2.html (accessed on 10 October 2025)) following the method described by Love et al. [28].

Gene Ontology (GO) enrichment analysis was performed to identify biological processes, cellular components, and molecular functions significantly associated with DEGs. GO terms with adjusted *p*-value (*p*-adjust) < 0.05 (Benjamini–Hochberg correction) were considered significantly enriched. KEGG pathway enrichment analysis was conducted to identify metabolic and signaling pathways significantly affected by ploidy, with corrected *p*-value < 0.05 as the significance threshold.

### 2.7. Quantitative Real-Time PCR (qRT-PCR) Validation

To validate the RNA-seq results, 12 genes related to gonadal development were selected for qRT-PCR analysis. First-strand cDNA was synthesized from 1 μg of total RNA using the Thermo Scientific RT Reverse Transcription Kit (Thermo Fisher Scientific, 154402, Waltham, MA, USA) according to the manufacturer’s instructions. qRT-PCR was performed on a Roche LightCycler 480 II instrument (Roche, Mannheim, Germany) using SYBR Green Premix (Yeasen, 11201ES03, Shanghai, China). Each reaction was run in triplicate. The *S. maximus β-actin* gene was used as an internal reference for normalization. Gene-specific primers (Table 1) were designed using NCBI Primer-BLAST (https://blast.ncbi.nlm.nih.gov/Blast.cgi (accessed on 15 December 2025)) and synthesized by Sangon Biotech Co., Ltd. (Shanghai, China). Relative gene expression levels were calculated using the 2^−ΔΔCt^ method [29].

### 2.8. Statistical Analysis

Data are presented as the mean ± standard deviation (SD). Statistical analyses were performed using SPSS 27.0 software (IBM, USA) for qRT-PCR data and R software (v4.2.0) for RNA-seq data. For RNA-seq data, differential expression analysis was performed using DESeq2 (v1.42.0) with a negative binomial generalized linear model, incorporating ploidy (diploid vs. triploid) and developmental stage (6, 10, and 20 mph) as factors. Genes with false discovery rate (FDR) < 0.05 and |log_2_ fold change| ≥ 2 were considered significantly differentially expressed. For qRT-PCR validation data, a two-way analysis of variance (two-way ANOVA) was used to assess the effects of ploidy (diploid vs. triploid), developmental stage (6, 10, and 20 mph), and their interaction. Tukey’s multiple comparison test was applied for post hoc comparisons between ploidy groups at the same developmental stage and between developmental stages within the same ploidy group. One-way ANOVA followed by Duncan’s post hoc test was used for comparisons across developmental stages within each ploidy group when analyzing the expression patterns of individual genes. Differences were considered statistically significant at *p* < 0.05.

## 3. Results

### 3.1. Ploidy Confirmation

**Figure 1:**
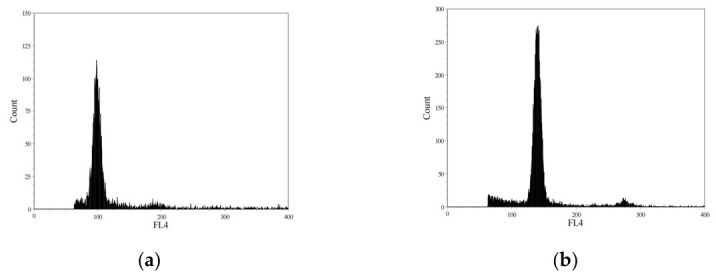
Representative DNA content histograms of diploid (**a**) and triploid (**b**) turbot at 20 mph. X-axis: fluorescence (FL) values on an arbitrary scale. The triploid sample shows a peak at approximately 1.5 times the fluorescence intensity of the diploid control.

Flow cytometric analysis confirmed that all triploid individuals used in this study maintained a stable triploid status throughout development. At each sampling stage (6, 10, and 20 mph), triploid fish exhibited a relative DNA content approximately 1.5-fold higher than that of diploid controls (Figure 1). Only fish confirmed as triploid were used for subsequent histological and transcriptomic analyses.

### 3.2. Histological Observations of Gonadal Development in Diploid and Triploid

**Figure 2:**
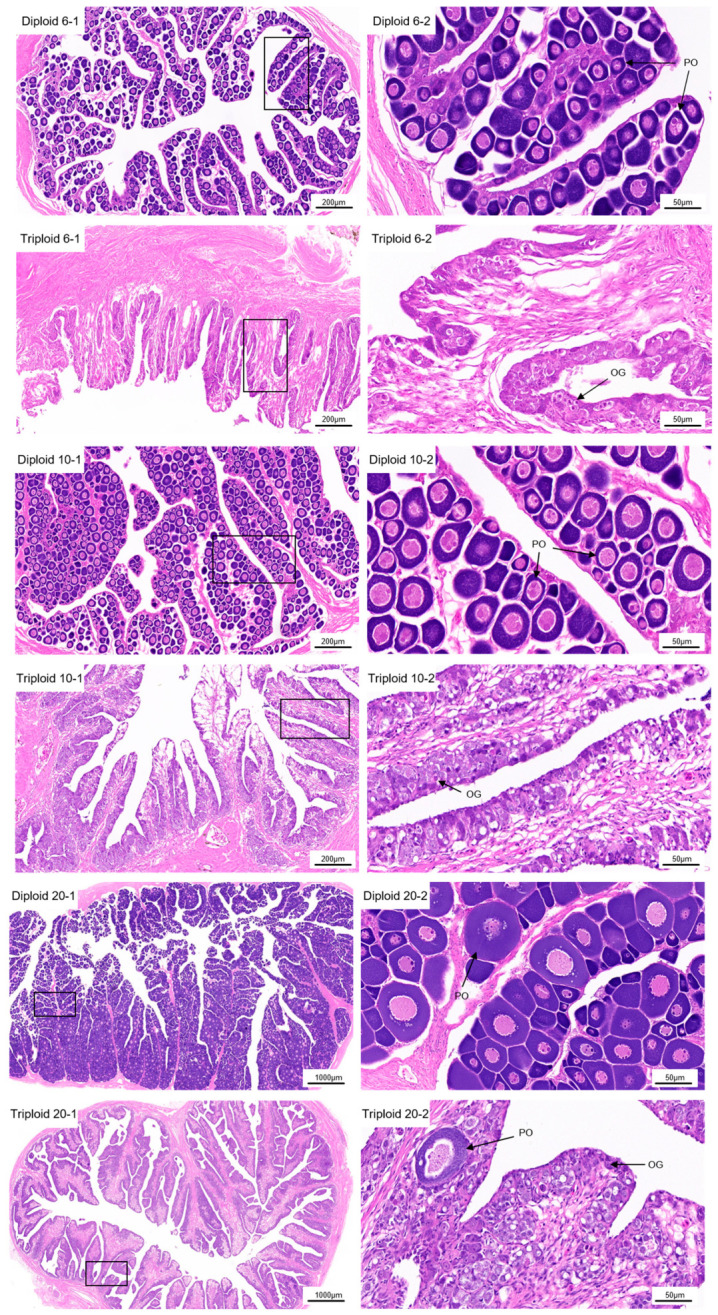
Histology of the ovaries in diploid and triploid turbot at 6, 10, and 20 months post-hatch (mph). For each developmental stage and ploidy, two magnifications are shown: the left panels with suffix “-1” are low-magnification overviews; the right panels with suffix “-2” are higher-magnification views of the boxed regions in the corresponding “-1” panels. Images are directly labeled as “Diploid” (6, 10, 20) and “Triploid” (6, 10, 20) on the panels for ease of reference. 6-1 (overview) and 6-2 (boxed region) at 6 mph; 10-1 and 10-2 at 10 mph; 20-1 and 20-2 at 20 mph. Oocyte stages are defined according to previous turbot studies: phase I, primary oocytes (perinucleolar stage); phase II, previtellogenic oocytes; phase III, early vitellogenic oocytes. PO: primary oocyte (phase I); OG: oogonia.

Histological analysis revealed marked differences in ovarian development between diploid and triploid *S. maximus* across all three developmental stages (Figure 2).

At 6 mph, diploid ovaries were in the proliferative stage, characterized by an abundance of primary oocytes (phase I, perinucleolar stage) distributed throughout the ovarian lamellae. These oocytes exhibited highly condensed chromatin within the nucleus (Figure 2 Diploid 6-1, Diploid 6-2). In contrast, triploid ovaries at the same stage were predominantly composed of connective tissue, with germ cells consisting almost exclusively of oogonia (Figure 2 Triploid 6-1, Triploid 6-2). No primary oocytes were observed in triploid ovaries at this stage.

At 10 mph, diploid ovaries had progressed to the previtellogenic stage (phase II), characterized by oocyte growth, enlargement of the germinal vesicle, and the appearance of multiple nucleoli at the periphery of the nucleus (Figure 2 Diploid 10-1, Diploid 10-2). By contrast, triploid ovaries remained largely undeveloped, with no evidence of oocyte progression beyond the oogonial stage. The ovarian structure was dominated by stromal tissue, and only occasional oogonia were observed (Figure 2 Triploid 10-1, Triploid 10-2).

At 20 mph, diploid ovaries had entered the early vitellogenic stage (phase III), characterized by the centralized appearance of spherical, eosinophilic, and vitellogenic yolk granules/globules within the oocyte cytoplasm (Figure 2 Diploid 20-1, Diploid 20-2). Phase III oocytes (early vitellogenic oocytes with evident yolk granule accumulation) predominated, showing a marked increase in cell volume. In triploid ovaries, however, only a limited number of phase I oocytes (primary oocytes at the perinucleolar stage) were detected, and these showed no developmental progression compared to earlier stages. The overall ovarian structure appeared atrophic, with extensive connective tissue infiltration and no evidence of vitellogenic oocytes (Figure 2 Triploid 20-1, Triploid 20-2).

### 3.3. Transcriptome Sequencing and Assembly

A total of 18 cDNA libraries were constructed from ovarian tissues of diploid and triploid *S. maximus* at 6, 10, and 20 mph, with three biological replicates per group. Paired-end sequencing generated between 37.3 and 55.8 million clean reads per library, corresponding to 5.61–8.28 Gb of clean data (Table 2). Quality assessment showed that Q20 exceeded 99% and Q30 exceeded 95% for all samples, indicating high base-calling accuracy. GC content ranged from 49.9% to 54.1%, with no significant GC bias observed. Mapping rates against the *S. maximus* reference genome ranged from 96.3% to 97.0%, demonstrating high-quality sequencing data suitable for downstream analysis.

**Figure 3:**
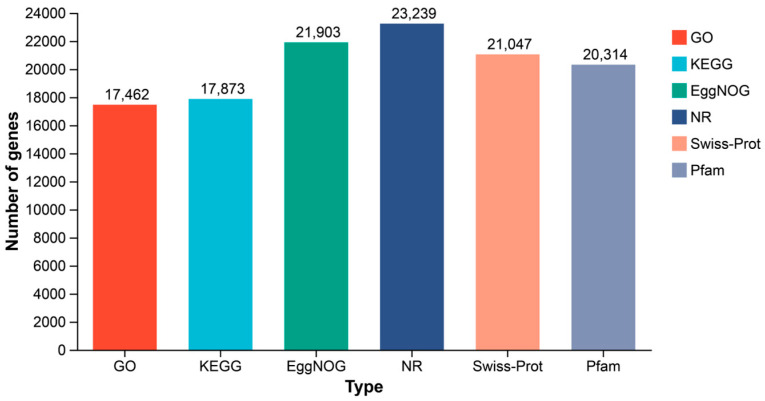
Annotation of genes in different databases. The X-axis represents the names of databases; the Y-axis represents the number of genes annotated to the corresponding database.

Functional annotation of assembled transcripts was performed against multiple public databases. Of 24,015 successfully annotated genes, 17,462 (72.7%) were annotated in the GO database, 17,873 (74.4%) in KEGG, 21,903 (91.2%) in EggNOG, 23,239 (96.8%) in NR, 21,047 (87.6%) in Swiss-Prot, and 20,314 (84.6%) in Pfam (Figure 3 and Table 3), indicating comprehensive coverage of the transcriptome.

### 3.4. Identification of Differentially Expressed Genes

**Figure 4:**
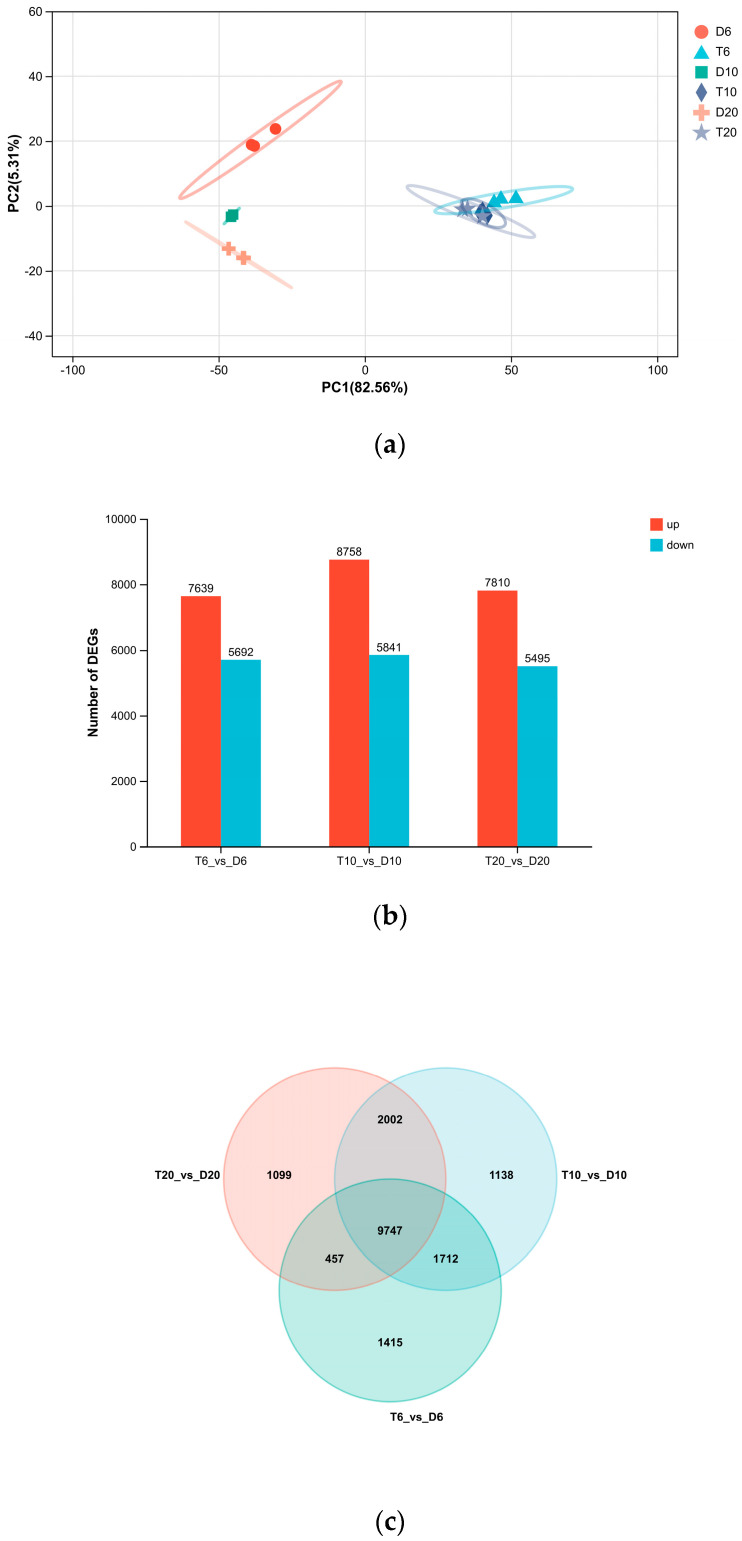
Comparative Analysis of DEGs. Separation of diploid and triploid ovaries of turbot by the principal component (PC) analysis (**a**). Numbers of DEGs of ovaries transcriptome at different development stages in turbot (**b**). Venn diagram illustrating the overlap of DEGs among the three comparison groups (**c**).

Principal component analysis (PCA) revealed clear separation between diploid and triploid samples along the principal components, while biological replicates within each ploidy group clustered closely together (Figure 4a), demonstrating high reproducibility and distinct transcriptomic profiles between ploidies.

Pairwise comparison between triploid and diploid ovaries identified 13,305 DEGs at 6 mph (7810 upregulated, 5495 downregulated), 14,599 DEGs at 10 mph (8758 upregulated, 5841 downregulated), and 13,331 DEGs at 20 mph (7639 upregulated, 5692 downregulated) (Figure 4b). Venn diagram analysis showed that 9747 DEGs were common across all three stages, while 11,459, 10,204, and 11,749 DEGs were shared between the 6 and 10 mph, 6 and 20 mph, and 10 and 20 mph comparisons, respectively (Figure 4c), indicating both conserved and stage-specific transcriptomic responses to triploidy.

Key gonadal development-related DEGs identified in the ovaries, including those involved in meiosis (*spo11*, *dmc1*, *rad51*, *sycp3*), oogenesis (*gdf9*, *bmp15*, *pou5f3*), sex differentiation (*rspo1*, *wnt4*, *foxl2a*, *sox9*, *ctnnb1*), energy metabolism (*cox5a*, *sdha*, *ndufa11*, *prkaa2*), and apoptosis (*eif2ak3*, *apaf1*, *bcl2b*), are listed in Table 4. Notably, while rspo1 was significantly upregulated in triploid ovaries across all stages (6 mph: log_2_FC = 6.13, *p* = 3.13 × 10^−19^; 10 mph: log_2_FC = 4.30, *p* = 1.70 × 10^−14^; 20 mph: log_2_FC = 2.56, *p* = 2.14 × 10^−4^), its expression level showed a progressive decline over developmental time, suggesting a diminishing transcriptional response as triploid ovaries become developmentally arrested.

### 3.5. GO Enrichment Analysis of DEGs

**Figure 5:**
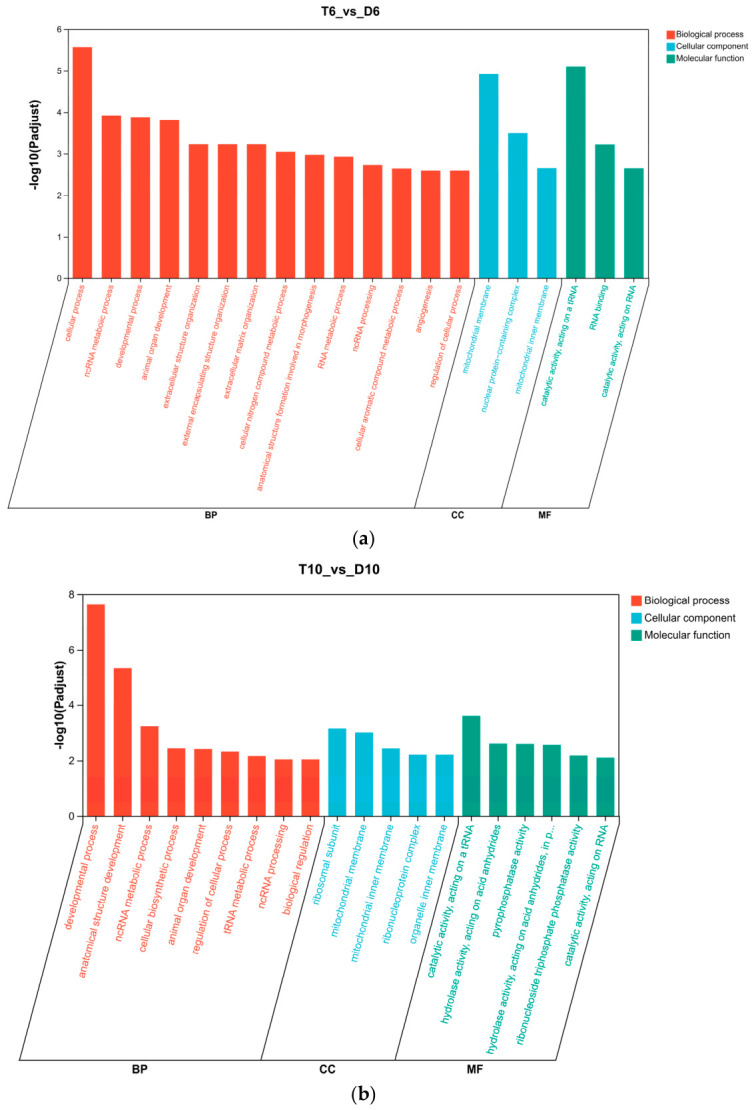

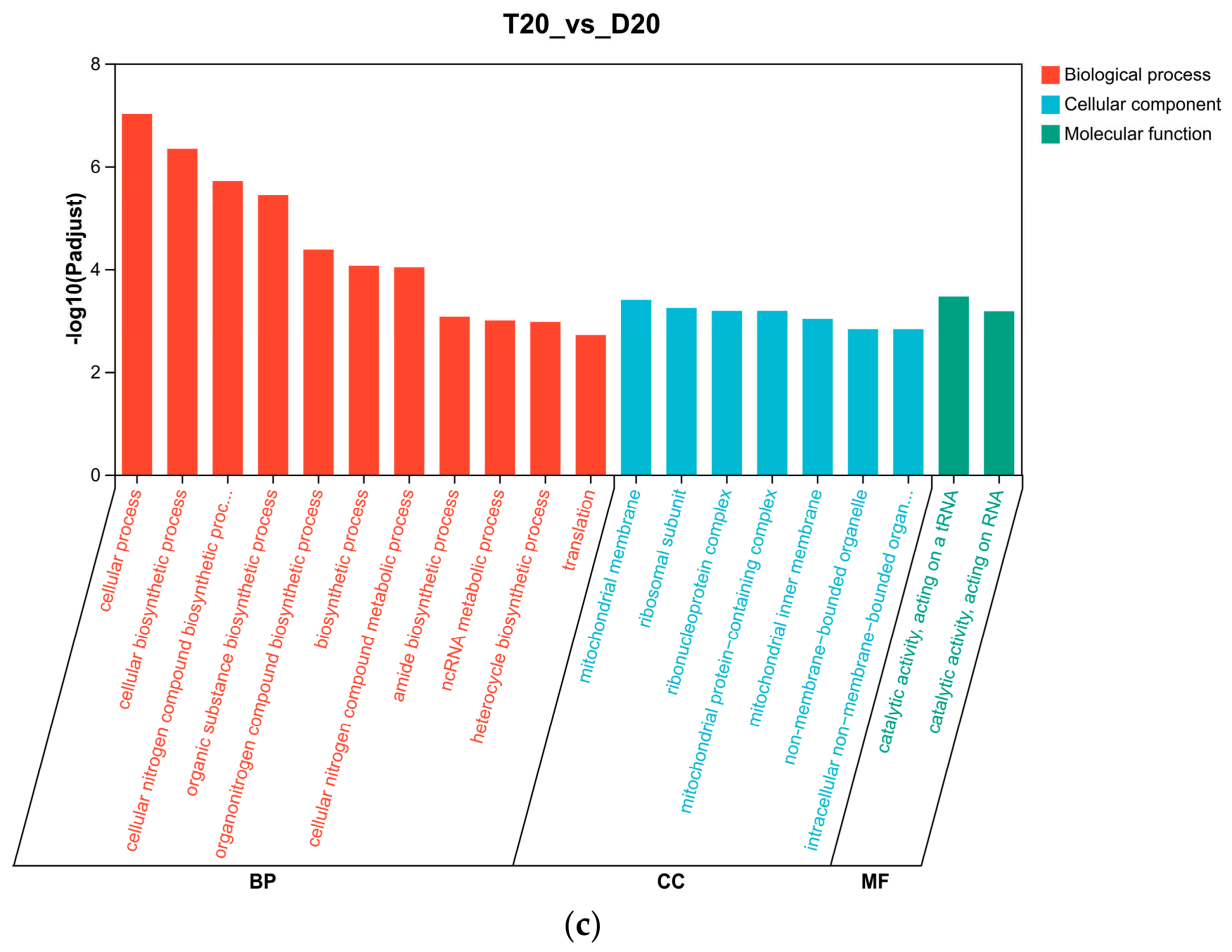
Gene Ontology (GO) functional enrichment analysis of DEGs: (**a**) T6 vs. D6; (**b**) T10 vs. D10; (**c**) T20 vs. D20. Only GO terms with adjusted *p*-value (*p*-adjust) < 0.05 (Benjamini–Hochberg correction) are shown. Color intensity represents the adjusted *p*-value, with darker colors indicating greater significance.

Gene Ontology (GO) enrichment analysis was performed to elucidate the biological functions of DEGs between diploid and triploid ovaries across the three developmental stages (Figure 5).

At 6 mph, the 13,305 DEGs were significantly enriched in 353 GO terms, comprising 214 biological processes (BP), 53 cellular components (CC), and 86 molecular functions (MF). Among these, terms related to gonadal development—including animal organ development (GO:0032502) and cell differentiation (GO:0009987)—were prominently enriched (Figure 5a). The most significantly enriched CC categories included mitochondrial membrane and ribonucleoprotein complex, while MF categories were dominated by catalytic activities acting on RNA and tRNA.

At 10 mph, 14,599 DEGs were mapped to 334 GO terms (188 BP, 59 CC, 87 MF). Significant enrichment was observed in BP terms such as cellular biosynthetic process (GO:0044249) and cell differentiation (GO:0009987), indicating sustained disruption of fundamental cellular processes in triploid ovaries (Figure 5b). The CC and MF enrichment patterns were similar to those observed at 6 mph, with mitochondrial-related components and catalytic activities remaining prominently enriched.

At 20 mph, 13,331 DEGs were assigned to 347 GO terms (222 BP, 52 CC, 73 MF). Significantly enriched BP terms included cellular process (GO:0009987), cellular biosynthetic process (GO:0044249), and biosynthetic process (GO:0009058) (Figure 5c). Across all three stages, DEGs were predominantly enriched in BP categories related to cellular processes, metabolic processes, and developmental processes, suggesting that triploidy induces widespread and persistent transcriptional dysregulation in ovarian tissues.

### 3.6. KEGG Pathway Enrichment Analysis of DEGs

**Figure 6:**
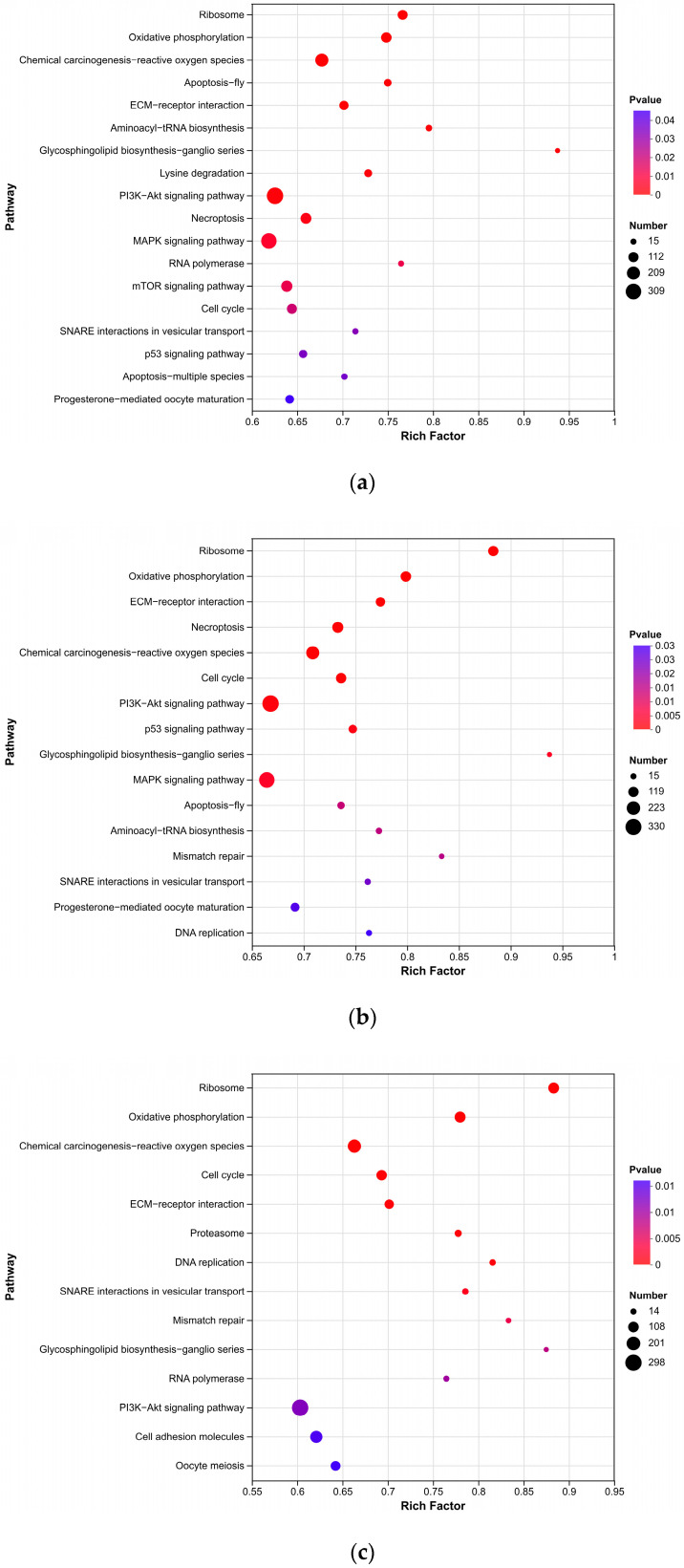
KEGG pathway enrichment analysis of DEGs: (**a**) T6 vs. D6; (**b**) T10 vs. D10; (**c**) T20 vs. D20. Only pathways with corrected *p*-value < 0.05 are shown. Color intensity represents the corrected *p*-value, with darker colors indicating greater significance.

To identify the specific biological pathways affected by triploidy, KEGG pathway enrichment analysis was performed on DEGs identified at each developmental stage (Figure 6).

At 6 mph, DEGs were mapped to 359 pathways, with significant enrichment observed in pathways related to gonadal development and cellular metabolism, including Ribosome, Progesterone-mediated oocyte maturation, Aminoacyl-tRNA biosynthesis, and ECM-receptor interaction (Figure 6a). The enrichment of Ribosome and Aminoacyl-tRNA biosynthesis pathways indicated dysregulation of protein synthesis machinery in triploid ovaries at this early stage.

At 10 mph, DEGs were enriched in 369 pathways, with Progesterone-mediated oocyte maturation, ECM-receptor interaction, and Cell cycle emerging as the most significantly affected pathways related to reproduction and cellular proliferation (Figure 6b). The enrichment of Cell cycle pathway suggested disruption of cell division processes during the critical previtellogenic transition.

At 20 mph, DEGs were mapped to 356 pathways, with significant enrichment in Cell cycle, DNA replication, and Oocyte meiosis (Figure 6c). These pathways are directly involved in meiotic progression and oocyte development, consistent with the arrested oogenesis phenotype observed in triploid ovaries.

Across all three stages, several key signaling and metabolic pathways—including Oxidative phosphorylation, PI3K-Akt signaling pathway, MAPK signaling pathway, and Necroptosis—were consistently enriched, indicating that triploidy affects core cellular functions such as energy metabolism, cell survival, and programmed cell death throughout ovarian development.

Notably, stage-specific shifts in pathway enrichment were observed. In early stages (6–10 mph), pathways related to apoptosis (Apoptosis), stress response (MAPK signaling pathway), and DNA damage (p53 signaling pathway) were predominantly enriched, suggesting active elimination of aberrant germ cells during meiotic initiation. In contrast, at 20 mph, enrichment shifted toward Proteasome and Cell adhesion molecules, indicating a transition from active clearance to maintenance of cellular homeostasis in the developmentally arrested triploid ovaries.

### 3.7. Validation of RNA-Seq Data by qRT-PCR

**Figure 7:**
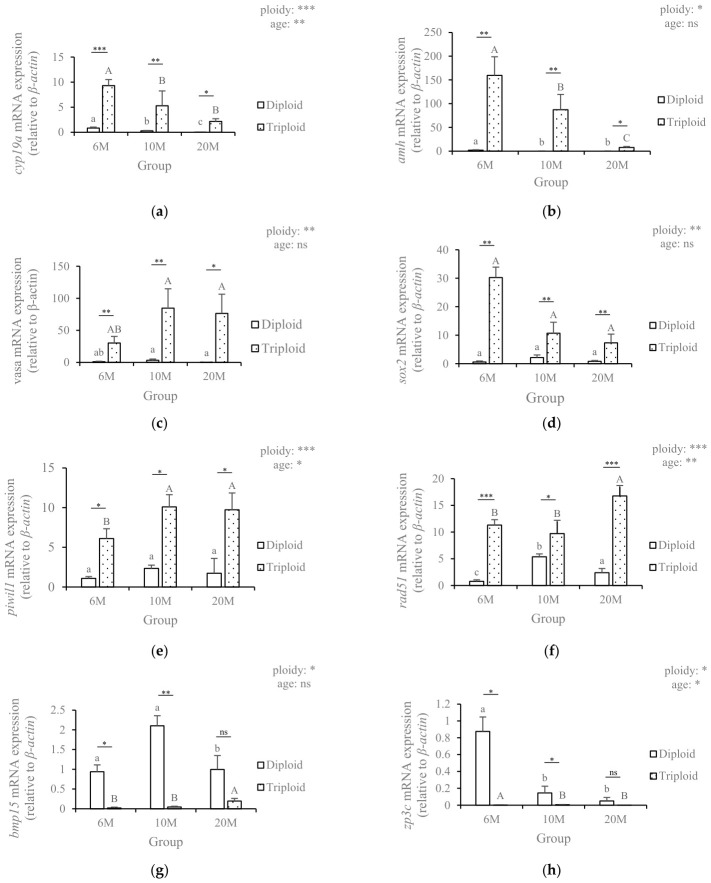

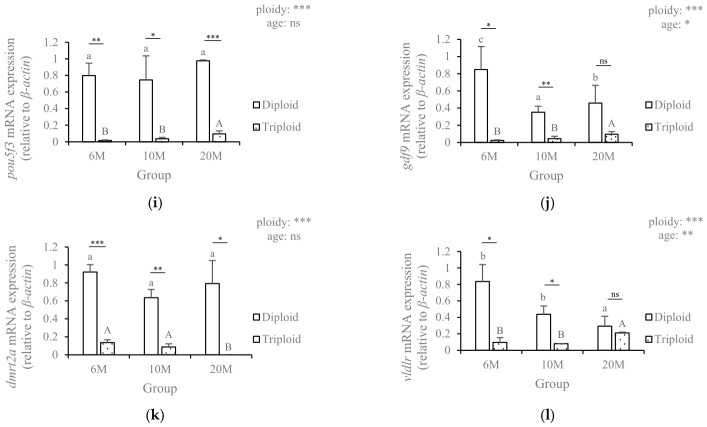
Validation of 12 differentially expressed genes by qRT-PCR. (**a**) *cyp19a* mRNA expression; (**b**) *amh* mRNA expression; (**c**) *vasa* mRNA expression; (**d**) *sox2* mRNA expression; (**e**) *piwil1* mRNA expression; (**f**) *rad51* mRNA expression; (**g**) *bmp15* mRNA expression; (**h**) *zp3c* mRNA expression; (**i**) pou5f3 mRNA expression; (**j**) *gdf9* mRNA expression; (**k**) *dmrt2a* mRNA expression; (**l**) *vldlr* mRNA expression. Different lowercase letters (a, b, c) indicate significant differences across developmental stages within the diploid group (one-way ANOVA followed by Duncan’s post hoc test, *p* < 0.05). Different uppercase letters (A, B, C) indicate significant differences across developmental stages within the triploid group (one-way ANOVA followed by Duncan’s post hoc test, *p* < 0.05). Asterisks above the bars indicate significant differences between diploid and triploid groups at the same developmental stage (one-way ANOVA followed by Tukey’s multiple comparison test: * *p* < 0.05; ** *p* < 0.01; *** *p* < 0.001; ns, no significant difference). Asterisks in the upper right corner indicate the significance levels of the main effects of ploidy and developmental stage, as determined by two-way ANOVA, using the same significance notation.

To validate the reliability of the RNA-seq data, 12 genes involved in gonadal development, meiosis, and apoptosis were selected for qRT-PCR analysis. The expression patterns of all selected genes, including *cyp19a*, *amh*, *vasa*, *sox2*, *piwil1*, *rad51*, *bmp15*, *zp3c*, *pou5f3*, *gdf9*, *dmrt2a* and *vldlr*, showed consistent trends between qRT-PCR and RNA-seq data (Figure 7). Statistical analysis using two-way ANOVA demonstrated that ploidy exerted significant effects on the expression of all detected genes. For developmental stage, significant differences were observed in the expression of *cyp19a*, *piwil1*, *rad51*, *zp3c*, *gdf9* and *vldlr*, whereas no statistically significant differences were detected in the remaining genes (ns). These findings collectively validate the robustness and accuracy of the transcriptomic analysis.

## 4. Discussion

### 4.1. Histological Evidence of Arrested Oogenesis in Triploid Turbot

The present study provides histological evidence that ovarian development in triploid *S. maximus* is severely impaired across all examined stages, consistent with observations in other triploid teleosts. At 6 mph, diploid ovaries contained abundant primary oocytes, indicating successful entry into meiotic prophase, whereas triploid ovaries were dominated by oogonia and connective tissue with no detectable primary oocytes. This aligns with findings in triploid *O*. *mykiss*, where primary oocytes were absent in females [14], and in triploid *P*. *olivaceus*, where ovarian development was severely impaired with atrophy and testicularization [16].

By 10 mph, diploid ovaries had progressed to the previtellogenic stage, characterized by oocyte growth and germinal vesicle enlargement, while triploid ovaries remained arrested at the oogonial stage. At 20 mph, diploid ovaries entered early vitellogenesis with abundant yolk granules, whereas triploid ovaries contained only a limited number of phase I oocytes that failed to undergo further development, resulting in an overall atrophic appearance. This developmental arrest pattern mirrors reports in triploid *H*. *fossilis*, where oocyte development was arrested with reduced ovarian volume [15], and in triploid blunt snout bream *Megalobrama amblycephala*, where oocytes failed to develop beyond stage II [30]. These observations confirm that sterility in female triploid *S*. *maximus*, as in other species, is fundamentally rooted in meiotic failure and subsequent arrest of oogenesis.

### 4.2. Transcriptional Dysregulation Underlying Meiotic Failure

Transcriptomic analysis revealed widespread gene expression changes in triploid ovaries, with over 13,000 DEGs identified at each developmental stage. Among these, key meiotic genes exhibited distinct expression patterns that help explain the observed arrest. Notably, the significant upregulation of genes involved in meiotic initiation and progression—including *spo11* (meiotic recombination), *dmc1* (homologous pairing), *sycp3* (synaptonemal complex formation), and *msh4*/*5* (crossover formation)—indicates that triploid germ cells retain the intrinsic developmental program and attempt to initiate meiosis. However, the histological absence of primary oocytes suggests that this attempted entry fails to progress beyond the earliest stages of meiotic prophase. This failure can be attributed to two interrelated mechanisms.

First, the presence of three chromosome sets is predicted to disrupt the one-to-one correspondence required for homologous pairing, potentially leading to a chaotic ‘one-to-many’ pairing pattern. Second, and critically, *rad5*, a core factor in homologous recombination repair, was significantly downregulated in triploid ovaries, showing a striking divergence from the upregulation of *dmc1*. In teleosts, Dmc1 and Rad51 play distinct roles in meiotic recombination, and their coordinated activity is essential for proper strand invasion and homology search. This *dmc1*–*rad51* expression imbalance likely disrupts the normal coordination of meiotic recombinases, leading to aberrant meiotic initiation that triggers checkpoint-mediated apoptosis. Consequently, germ cells that attempt to enter meiosis are eliminated before reaching histologically recognizable meiotic stages (leptotene, zygotene), resolving the apparent paradox between transcriptional activation and developmental arrest. Such regulatory crosstalk between meiotic recombinases has been documented in other systems, where Sycp3 suppresses RAD51-mediated strand invasion while preserving *dmc1* activity [31], and where negative feedback loops coordinate double-strand break repair with meiotic progression [32]. Whether the observed *dmc1*–*rad51* expression imbalance in triploid *S*. *maximus* reflects a similar regulatory mechanism, or represents a consequence of aberrant meiotic progression, warrants further investigation.

Furthermore, regulators of meiotic entry, including *cyp26b1* and *nanos2*, which control retinoic acid signaling and germ cell fate, respectively [33,34,35], showed dysregulated expression in triploid ovaries. The imbalance in these regulatory factors likely exacerbates meiotic disorders, contributing to the developmental arrest observed.

We acknowledge that bulk RNA-seq analysis of whole ovarian tissue cannot fully distinguish between changes in cell-autonomous gene expression and shifts in relative cell type abundance. The reduced proportion of germ cells and increased stromal/connective tissue in triploid ovaries may contribute to the observed differential expression of certain genes. However, the consistent enrichment of meiosis- and oogenesis-specific pathways across developmental stages, coupled with the concordance between transcriptional and histological evidence of apoptosis, supports the interpretation that the DEGs reflect genuine dysregulation of biological processes within the ovarian cell populations, rather than being solely attributable to altered cellular composition. Future studies employing single-cell transcriptomics or spatial profiling will be valuable for resolving cell-type-specific expression changes in triploid gonads.

### 4.3. Impaired DNA Repair and Cell Cycle Progression

The DNA mismatch repair (MMR) system and replication factor complexes play essential roles in maintaining genomic stability during meiosis [36,37,38]. In the present study, significant downregulation of *msh2*, *msh3*, and *msh6* was observed in triploid ovaries, suggesting ineffective repair of mismatched bases and insertion/deletion loops during meiotic recombination. This deficiency likely triggers oocyte apoptosis as a quality control mechanism. Similarly, downregulation of replication factor C genes (*rfc1*, *rfc2*, *rfc3*, *rfc5*) indicates disrupted DNA replication and cell cycle checkpoint control, further contributing to meiotic failure.

The enrichment of Cell cycle and DNA replication pathways in KEGG analysis, particularly at 10 and 20 mph, corroborates these findings. Comparable results have been reported in triploid *O*. *mykiss*, where ovarian transcriptome analysis revealed dysregulation of cell cycle-related pathways [39]. The persistence of these disruptions across all developmental stages suggests that triploidy-induced genomic instability is a continuous rather than transient phenomenon.

### 4.4. Disruption of Oogenesis Regulatory Networks

Oogenesis is orchestrated by a complex network of genes regulating follicular development and oocyte maturation. In triploid *S. maximus* ovaries, significant downregulation of key oogenic regulators—including *pou5f3* (germ cell pluripotency), *gdf9* (oocyte maturation), and *bmp15* (follicle development)—is inconsistent with the histological phenotype of failed oocyte formation. These findings align with studies in zebrafish *Danio rerio* where *bmp15* knockout resulted in later-stage ovarian-to-testicular reversal [40,41], and in mice *Mus musculus* where *gdf9* and *smad2* disruption impaired oocyte maturation [42,43].

Additionally, genes involved in nutrient accumulation—particularly members of the Low-Density Lipoprotein Receptor family (*lr8*, *lrp13*, *vldlr*)—were significantly downregulated in triploid ovaries. This likely compromises the oocyte’s ability to acquire yolk precursors, further inhibiting maturation [44]. The combined downregulation of pluripotency maintenance, oocyte growth, and nutrient uptake genes provides a molecular explanation for the arrested oogenesis observed histologically.

### 4.5. Dysregulation of the RSPO1/WNT/β-Catenin Signaling Pathway

The RSPO1/WNT/β-catenin pathway is a conserved regulator of vertebrate sex determination, though its role in teleosts extends beyond ovarian differentiation. In medaka *Oryzias latipes*, rspo1 overexpression induces complete sex reversal in XY individuals [45], while in Nile tilapia *Oreochromis niloticus*, *rspo1* knockout impairs both ovarian development and spermatogenesis, demonstrating its dual requirement in teleost gonadal function [46].

In the present study, *rspo1* and *wnt4* were significantly upregulated in triploid *S. maximus* ovaries across all stages, whereas the expression of β-catenin (encoded by *ctnnb1*) was downregulated. This uncoupling of ligand upregulation from core pathway activation is a notable finding, though its interpretation is constrained by the lack of cell-type-specific expression data, as bulk RNA-seq cannot distinguish whether these changes occur in germ cells, somatic cells, or both. Several factors may account for this apparent discrepancy. First, Rspo1 is a secreted protein whose activity depends not only on transcript abundance but also on post-translational regulation and the availability of co-receptors such as LGR family members; therefore, transcriptional upregulation of *rspo1* does not necessarily equate to increased pathway output. Second, as noted earlier, the RSPO1/WNT/β-catenin pathway exhibits a dual role in teleost gonadal function [45,46], and its dysregulation in the context of triploidy may manifest as an uncoordinated or abortive activation pattern rather than a coherent compensatory response. Interestingly, the magnitude of *rspo1* upregulation progressively declined from 6 mph (log_2_FC = 6.13) to 20 mph (log_2_FC = 2.56), suggesting that the initial strong transcriptional response to meiotic disruption attenuates over time. This decline may reflect the eventual stabilization of the triploid ovarian environment following the massive germ cell loss observed in early stages, transitioning from an acute stress response to a chronic homeostatic state. Alternatively, this expression pattern could reflect a partial of disorganized activation of testicular differentiation within the ovarian context, consistent with the “ovarian testicularization” phenotype reported in triploid *P*. *olivaceus* [16].

The disconnection between upstream ligand expression and canonical pathway activation suggests that RSPO1/WNT dysregulation is associated with arrested ovarian development in triploid *S. maximus*, but the precise mechanistic relationship requires further functional investigation, ideally employing cell-type-specific approaches.

### 4.6. Energy Metabolism Dysfunction and Mitochondrial Impairment

Oocyte maturation is an energy-intensive process requiring substantial ATP production to support vitellogenesis, meiosis resumption, and chromatin remodeling [47]. Mitochondria are the primary sites of ATP synthesis, and their function depends on proper expression of respiratory chain genes [48]. In triploid *S. maximus* ovaries, significant downregulation of nuclear-encoded mitochondrial genes (*acaa2*, *ndufa11*, *sdha*, *ndufs3*) was observed, indicating severe mitochondrial dysfunction.

This finding aligns with reports in triploid *C*. *carpio*, where mitochondrially encoded genes were generally downregulated in testes [31], and in triploid *C*. *gigas*, where energy metabolism pathways were significantly altered [49]. In addition, genes involved in AMPK signaling, including *prkaa2* (encoding the α2 subunit of AMP-activated protein kinase), were dysregulated in triploid ovaries. Given that AMPK activity is primarily regulated at the post-translational level [50,51,52,53], the functional significance of *prkaa2* transcriptional changes remains unclear. Nevertheless, the broader dysregulation of energy metabolism pathways observed in triploid ovaries likely contributes to the metabolic constraints on oocyte development. Consequently, insufficient ATP production likely prevents normal lipid transport to oocytes, which could contribute to impaired vitellogenesis and the developmental abnormalities observed histologically.

### 4.7. Apoptosis as a Mechanism of Germ Cell Elimination

Cell apoptosis is a well-established mechanism contributing to reproductive disorders in triploid fish, particularly in females [23]. Direct histological evidence of elevated germ cell apoptosis in triploid *S. maximus* ovaries has been previously documented by TUNEL staining, confirming the occurrence of programmed cell death in this species [23]. In the present study, while we did not perform direct in situ apoptosis detection, our transcriptomic data provide molecular insights consistent with this established phenotype. Mitochondrial dysfunction is a potent inducer of apoptosis: downregulation of mitochondrial genes disrupts the respiratory chain, leading to cytochrome c release and activation of caspases [54,55,56]. In triploid *S. maximus* ovaries, significant upregulation of apoptotic genes (*casp8*, *apaf1*) and ubiquitination-related genes (*ube4a*, *rnf2*) was observed alongside mitochondrial dysfunction.

Mitochondrial impairment can induce both apoptosis and aberrant activation of ubiquitin-mediated proteolysis, ultimately leading to massive germ cell loss. This cascade represents a critical molecular mechanism underlying ovarian developmental abnormalities in triploids. The enrichment of apoptotic pathways in early stages (6–10 mph) suggests that active elimination of aberrant germ cells occurs during meiotic initiation, while at later stages, the remaining arrested oocytes may persist in a state of cellular stress without undergoing active apoptosis.

### 4.8. Stage-Specific Shifts in Signaling Pathway Activation

KEGG pathway enrichment analysis revealed both persistent and stage-specific alterations in signaling pathways underlying triploid sterility. Across all developmental stages, pathways related to core cellular functions—including Ribosome, Oxidative phosphorylation, ECM-receptor interaction, PI3K-Akt signaling, and Cell cycle—were consistently enriched, indicating that triploidy induces sustained dysregulation of protein synthesis, energy metabolism, and cell proliferation machinery in ovarian tissues.

The PI3K-Akt signaling pathway, a central regulator of cellular energy metabolism and survival, was significantly inhibited in triploid ovaries across all stages. Under normal conditions, PI3K-Akt activation promotes oocyte nutrient uptake and exerts anti-apoptotic effects [57,58]. Its suppression in triploids likely contributes to impaired oocyte growth and increased susceptibility to cell death, consistent with the observed downregulation of energy metabolism genes (*cox5a*, *sdha*, *ndufs3*, *ctsba*) and upregulation of apoptotic genes (*eif2ak3*, *apaf1*). This coordinated dysregulation creates a vicious cycle of energy deficit and germ cell loss.

Notably, stage-specific shifts in pathway enrichment were observed. In early stages (6–10 mph), pathways related to apoptosis, MAPK signaling, and p53 signaling were predominantly enriched. This pattern suggests that triploidy-induced chromosomal abnormalities trigger a strong DNA damage stress response during meiotic initiation. MAPK signaling, acting as an upstream sensor, likely activates the p53 tumor suppressor pathway, initiating programmed cell death to systematically eliminate germ cells with meiotic abnormalities [59,60]. This active clearance mechanism represents an early key event leading to gonadal developmental arrest.

By 20 mph, these stress-response pathways were no longer enriched; instead, the proteasome pathway was significantly activated. This shift indicates a fundamental change in the adaptive strategy of triploid ovaries. Following large-scale apoptotic elimination in early stages, ovarian development becomes arrested at the primary oocyte stage, and the remaining cell population transitions from active elimination to long-term survival. Proteasome activation likely serves to cope with accumulated abnormal proteins resulting from prolonged developmental arrest, maintaining basic cellular homeostasis through enhanced protein quality control [61,62].

This transition from stress-induced apoptosis to proteasome-mediated homeostasis maintenance reveals that triploid sterility is not a static condition but a dynamic, multi-stage process: developmental fate is determined through stringent germ cell screening in early stages, followed by metabolic adjustment and homeostasis preservation in later stages to adapt to irreversible developmental arrest.

## 5. Conclusions

The present study provides comprehensive histological and transcriptomic evidence that ovarian development in triploid *S. maximus* is severely arrested in conjunction with meiotic failure, mitochondrial dysfunction, energy metabolism imbalance, and apoptosis-mediated germ cell elimination. The stage-specific shifts from stress-apoptosis dominated pathways to homeostasis-proteasome dominated pathways reveal a dynamic adaptive process underlying triploid sterility.

These findings enhance our understanding of the molecular mechanisms governing sterility in female triploid fish and provide valuable insights for optimizing triploid production in aquaculture. The stage-specific expression patterns of meiotic (*sycp3*, *dmc1*), apoptotic (*apaf1*, *eif2ak3*), and proteasome-related genes identified in this study offer potential molecular markers for assessing triploid sterility at early developmental stages, enabling more efficient screening in triploid breeding programs. Future functional studies using gene editing in diploid models could elucidate the causal roles of these genes in normal oogenesis and inform strategies for reproductive control in aquaculture.

## Figures and Tables

**Table 1:** Primer nucleotide sequences used in this study.

Name	Primer Sequence (5′ to 3′)
GDF9_F	AAACCAGGACAGAGGTTCGG
GDF9_R	ACTCGTTGGGAACTCAGAG
VASA_F	GCTGCTGACTTCCTCAAGACGG
VASA_R	AAGACACCGCCCTCCCAGTATT
PIWIL1_F	GATAAGTGGGGACTGGGCTT
PIWIL1_R	ACCAGCCAGTTATTCAGCGG
BMP15_F	GTGTCGTCGGTGCTCTACTT
BMP15_R	CTGGAGGAAGAGGAGGTGGA
ZP3C_F	GCTTTAGATGCGTCCGGGAA
ZP3C_R	GTTGGAGTTGGGGTTTTGGC
CYP19A_F	TAGGCACAGCCAGCAACTAC
CYP19A_R	GCTCCCGAAACGTGAAGAGT
SOX2_F	TGGCCAGCTCCCAGGGCTA
SOX2_R	GGACGACGAGGTGACGACG
AMH_F	GACCACCCTGACTCCCCTC
AMH_R	CATCCCAATCTGCTCCACC
SOX8A_F	CGAAGACGCAAAAGCACCAA
SOX8A_R	GCCATCCCAGGTTCTGTCTT
DMRT2A_F	TATACCAGCGGCACATTCGT
DMRT2A_R	TGCTGTTCGGGAAGATGAGC
RAD51_F	CGCCCGAGCCTTCAACACA
RAD51_R	CACCACGGCAACGCCAAAC
VLDLR_F	GAGACCCTGACTGCAAGGAC
VLDLR_R	TTGCAGTTGACTTCATCGGA
POU5F3_F	GACCTTTTTCGCGTTCCCAC
POU5F3_R	TCTCGGTCTTGATTTCGGGC
β-actin_F	CATGTACGTTGCCATCCAAG
β-actin_R	ACCAGAGGCATACAGGGACA

**Table 2:** Summary of the Illumina sequencing of gonad transcriptomes in turbot.

Sample	Clean Reads	Clean Bases	Q20 (%)	Q30 (%)	GC Content (%)	Total Mapped (%)
D6_1	48,024,754	7,204,354,260	99.18	95.91	53.56	96.69
D6_2	40,714,940	6,111,019,173	99.21	95.93	54	96.93
D6_3	48,757,072	7,312,926,718	99.16	95.93	53.98	96.48
T6_1	42,816,144	6,430,288,794	99.12	95.73	51.06	96.25
T6_2	38,624,862	5,803,114,105	99.19	96.11	50.41	96.93
T6_3	47,326,384	7,116,040,505	99.16	95.9	50.99	96.72
D10_1	55,811,440	8,280,897,253	99.07	95.42	54.01	96.4
D10_2	48,081,880	7,145,513,023	99.22	96.19	54.05	96.47
D10_3	53,398,290	7,953,382,183	99.23	96.21	54.08	96.55
T10_1	39,408,386	5,925,710,419	99.23	96.26	49.91	96.43
T10_2	47,324,302	7,115,911,887	99.23	96.25	50.05	96.3
T10_3	49,598,488	7,458,836,546	99.24	96.3	49.9	96.34
D20_1	43,421,296	6,520,304,124	99.22	95.88	51.77	96.84
D20_2	50,228,318	7,543,874,507	99.26	96.09	52.93	96.99
D20_3	42,927,756	6,442,171,755	99.29	96.3	52.88	97.04
T20_1	44,107,650	6,623,803,003	99.16	95.91	50.6	96.47
T20_2	42,867,176	6,439,056,236	99.17	95.95	50.49	96.62
T20_3	37,325,348	5,607,781,855	99.15	95.79	50.2	96.56

D6, D10 and D20, females in the diploid groups at 6, 10 and 20 mph; T6, T10 and T20, females in the triploid groups at 6, 10 and 20 mph. The same below.

**Table 3:** Functional annotation of genes from turbot transcriptome.

Annotation Database	Number of Genes
GO	17,462
KEGG	17,873
EggNOG	21,903
NR	23,239
Swiss-prot	21,047
Pfam	20,314
All	24,015

GO—Gene Ontology; KEGG—Kyoto Encyclopedia of Gene and Genome; EggNOG—Evolutionary Genealogy of Genes: Non-supervised Orthologous Groups; NR—National Center for Biotechnology Information (NCBI) Refseq.

**Table 4:** Gonadal development-related differentially expressed genes (DEGs) and their expression dynamics in triploid vs. diploid turbot ovaries at different developmental stages. Positive log_2_FC values indicate upregulation in triploid compared to diploid ovaries; negative values indicate downregulation. Genes were considered differentially expressed with FDR < 0.05 and |log_2_FC| ≥ 2 (DESeq2).

Genes	T6 vs. D6		T10 vs. D10		T20 vs. D20	
	log_2_FC	*p*-Value	log_2_FC	*p*-Value	log_2_FC	*p*-Value
*spo11*	1.29	5.96 × 10^−6^	1.63	2.58 × 10^−31^	1.39	5.04 × 10^−9^
*dmc1*	6.62	6.71 × 10^−49^	7.40	7.92 × 10^−33^	8.43	1.38 × 10^−71^
*rad51*	−1.07	1.71 × 10^−6^	−1.18	1.26 × 10^−16^	−0.95	2.83 × 10^−8^
*sycp3*	5.89	1.35 × 10^−56^	7.42	8.75 × 10^−202^	8.31	8.63 × 10^−124^
*gdf9*	−6.08	4.60 × 10^−5^	−3.78	2.75 × 10^−88^	−3.61	7.44 × 10^−17^
*bmp15*	−4.32	3.88 × 10^−24^	−5.28	2.63 × 10^−239^	−3.33	1.38 × 10^−4^
*pou5f3*	−6.76	3.42 × 10^−53^	−5.76	3.23 × 10^−55^	−4.38	1.17 × 10^−17^
*rspo1*	6.13	3.13 × 10^−19^	4.30	1.70 × 10^−14^	2.56	2.14 × 10^−4^
*wnt4*	2.11	2.99 × 10^−3^	2.40	3.87 × 10^−14^	1.75	5.46 × 10^−5^
*foxl2a*	3.16	3.67 × 10^−2^	2.69	1.40 × 10^−29^	2.80	6.60 × 10^−11^
*sox9*	3.83	6.91 × 10^−18^	5.64	8.66 × 10^−20^	5.51	5.68 × 10^−24^
*cox* *5a*	−1.48	3.87 × 10^−8^	−1.61	3.21 × 10^−58^	−2.16	3.85 × 10^−38^
*sdha*	−1.31	2.91 × 10^−10^	−1.76	1.35 × 10^−128^	−1.50	2.04 × 10^−15^
*ndufa11*	−1.57	3.00 × 10^−6^	−1.35	1.00 × 10^−33^	−1.26	4.84 × 10^−10^
*prkaa2*	1.59	2.17 × 10^−6^	2.28	2.93 × 10^−21^	3.38	2.88 × 10^−21^
*eif2ak3*	2.28	1.10 × 10^−14^	2.08	1.41 × 10^−48^	1.55	5.59 × 10^−9^
*apaf1*	1.76	1.33 × 10^−6^	1.95	1.13 × 10^−24^	1.26	1.70 × 10^−5^
*bcl2b*	3.74	2.97 × 10^−13^	3.49	6.31 × 10^−14^	3.63	6.51 × 10^−10^
*ctnnb1*	−2.89	1.79 × 10^−16^	−2.83	2.00 × 10^−79^	−1.66	1.89 × 10^−15^

## Data Availability

All relevant data are within the paper and available from the corresponding author.
